# Using remote sensing to detect whale strandings in remote areas: The case of sei whales mass mortality in Chilean Patagonia

**DOI:** 10.1371/journal.pone.0222498

**Published:** 2019-10-17

**Authors:** Peter T. Fretwell, Jennifer A. Jackson, Mauricio J. Ulloa Encina, Vreni Häussermann, Maria J. Perez Alvarez, Carlos Olavarría, Carolina S. Gutstein

**Affiliations:** 1 British Antarctic Survey, Madingley Road, Cambridge, England, United Kingdom; 2 Aquatic Animal Rescue and Conservation Unit, National Fisheries and Aquaculture Service of Chile, Valparaiso, Chile; 3 Pontificia Universidad Católica de Valparaíso, Facultad de Recursos Naturales, Escuela de Ciencias del Mar, Avda. Brazil, Valparaíso, Chile; 4 Huinay Scientific Field Station, Valparaíso, Chile; 5 Escuela de Medicina Veterinaria, Facultad de Ciencias, Universidad Mayor, Santiago, Chile; 6 Instituto de Ecología y Biodiversidad, Facultad de Ciencias, Universidad de Chile, Santiago, Chile; 7 Centro de Investigación Eutropia, Santiago, Chile; 8 Centro de Estudios Avanzados en Zonas Aridas (CEAZA), La Serena, Chile; 9 Lab. Ontogenia y Filogenia, Depto. Biologiía, Fac. Ciencias, Universidad de Chile, Santiago, Chile; 10 Consultora Paleosuchus Ltda, oficina C, Providencia, Santiago, Chile; University of Guam, GUAM

## Abstract

We test the ability of Very High Resolution satellite (VHR) imagery to detect stranded whales using both manual and automated methods. We use the 2015 mass mortality event in the Gulf of Penas locality, central Patagonia, Chile, as an initial case study. This event was the largest known mass mortality of baleen whales, with at least 343 whales, mainly sei whales (Balaenoptera borealis), documented as stranding. However, even with such a large number of whales, due to the remote location of the gulf the strandings went unrecorded for several weeks. Aerial and boat surveys of the area were conducted two to four months after the mortality event. In this study we use 50cm resolution WorldView2 imagery to identify and count strandings from two archival images acquired just after the stranding event and two months before the aerial and ground surveys, and to test manual and automated methods of detecting stranded whales. Our findings show that whales are easily detected manually in the images but due to the heterogeneous colouration of decomposing whales, spectral indices are unsuitable for automatic detection. Our satellite counts suggest that, at the time the satellite images were taken, more whales were stranded than recorded in the aerial survey, possibly due to the non-comprehensive coverage of the aerial survey or movement of the carcases between survey acquisition. With even higher resolution imagery now available, satellite imagery may be a cost effective alternative to aerial surveys for future assessment of the extent of mass whale stranding events, especially in remote and inaccessible areas.

## Introduction

Temporal and spatial patterns of whale strandings (whales dead in the water and then beached) and Mass Mortality Events (MME) can be an important marker for temporal variability in oceanographic conditions [1]. The causes of marine mammal strandings are poorly understood [2], and therefore information gained from these events represents an important resource for understanding marine mammal health and diet [3], environmental pollution [4, 5, 6], regional oceanography [7–10], social structuring [11] and climate change [4, 12]. Strandings are also a useful proxy measure of species diversity, distribution and abundance [13–15]. Mass strandings have been variously attributed to navigation problems [particularly for odontocetes, e.g. [16]], unusual environmental conditions reducing prey availability [17], acute poisoning from harmful algal blooms (HAB) [10]and acoustic trauma [18–19]. Many coastal nations have mammal stranding networks, recognizing that this is a crucial means by which the health and welfare of local marine mammals can be monitored, and can provide first notice of potential marine contamination or pathogen spread [20].

Efficient detection of strandings is a major challenge in many areas, as resources tend to be concentrated on activities associated with carcass sampling and analysis, rather than on the initial detection. The scale of strandings can also be hard to identify, particularly in remote and inaccessible regions. In remote locations, Very High Resolution (VHR) remote sensing technology can offer a lower cost, large-scale means of monitoring for whale stranding events, permitting more rapid response times in order to identify and collect data on MMEs and improved understanding of the temporal and spatial extent of these events. Satellite technology has immense potential for the large-scale study of animal populations, even including the detection of live whales at sea [21–22].

At present the highest available spatial resolution of satellites is 31–50 cm in the panchromatic (grayscale) band and 1.2–2 m in the multispectral bands (visible colour/infrared). In remote areas this technology may prove more effective and economical than more traditional survey techniques such as ground, boat or aerial survey for assessing MMEs of large whales. Mass strandings of large baleen whales are much rarer than those of smaller odontocetes, and should receive particularly acute attention as they can signal unusual environmental conditions or Harmful Algal Bloom events, often with significant impacts on a swathe of less easily observable marine life (e.g. [22–25]. The sei whale (*Balaenoptera borealis*) stranding event in the Chilean Central Patagonia region in early 2015 was the largest baleen whale mass stranding ever recorded, with 343 primarily sei whales synchronously stranding along the coast between 46 and 48°S [24–26]. This coastline is extremely remote and extensively infolded, with many fjords, channels and islands. While Chile has a stranding response program (e.g., [27]), a systematic monitoring program covering these remote localities does not yet exist. The stranding event was discovered by scientists of Huinay Scientific Field Station conducting an unrelated expedition in April-May 2015. The stranding was then investigated via a vessel trip to part of the site (Estero Slight) in late May (25^th^-31^st^), and several follow up expeditions were carried out both from governmental (Sernapesca and Armada de Chile) and independent organizations (Huinay and Blue Marine Foundation). Subsequent to the original expedition, an aerial survey of the coast was conducted between June 23-27^th^ between Seno Newman (46°39’S) and the Jungfrauen Islands (48°S) in order to locate, count and photograph whale carcasses, and a satellite photo of 100 km^2^ from Seno Newman was taken on August 13, 2015. The Häussermann *et al*. [25] study also highlighted the potential use of satellite imagery to confirm the number of beached whales in Seno Newman (a small part of the survey area). This study used an image taken on 13^th^ of August, around five months after the peak of the strandings, and compared the image with locations ascertained in the aerial survey, and proposed that satellite imagery could be used to monitor whale strandings patterns in this region in future.

In this study, we further explore the use of VHR satellite images to survey the number and distribution of whales present at this mass stranding event in order to: (1) Quantify how well stranded whales can be identified and counted in Very High Resolution satellite imagery; (2) Explore if a simple spectral analysis could be used to automate the finding of stranded whales in satellite imagery; (3) Discuss the potential use of this technology in a wider global context.

This work is anticipated to represent a step towards methods that could be incorporated into a more automated system to acquire imagery, to monitor remote areas and provide an “early warning” of stranding events in places where strandings are regularly anticipated, i.e. where red tide toxins or other environmental factors indicate a high likelihood of stranding events.

## Materials and methods

### Area of study

**Fig 1:**
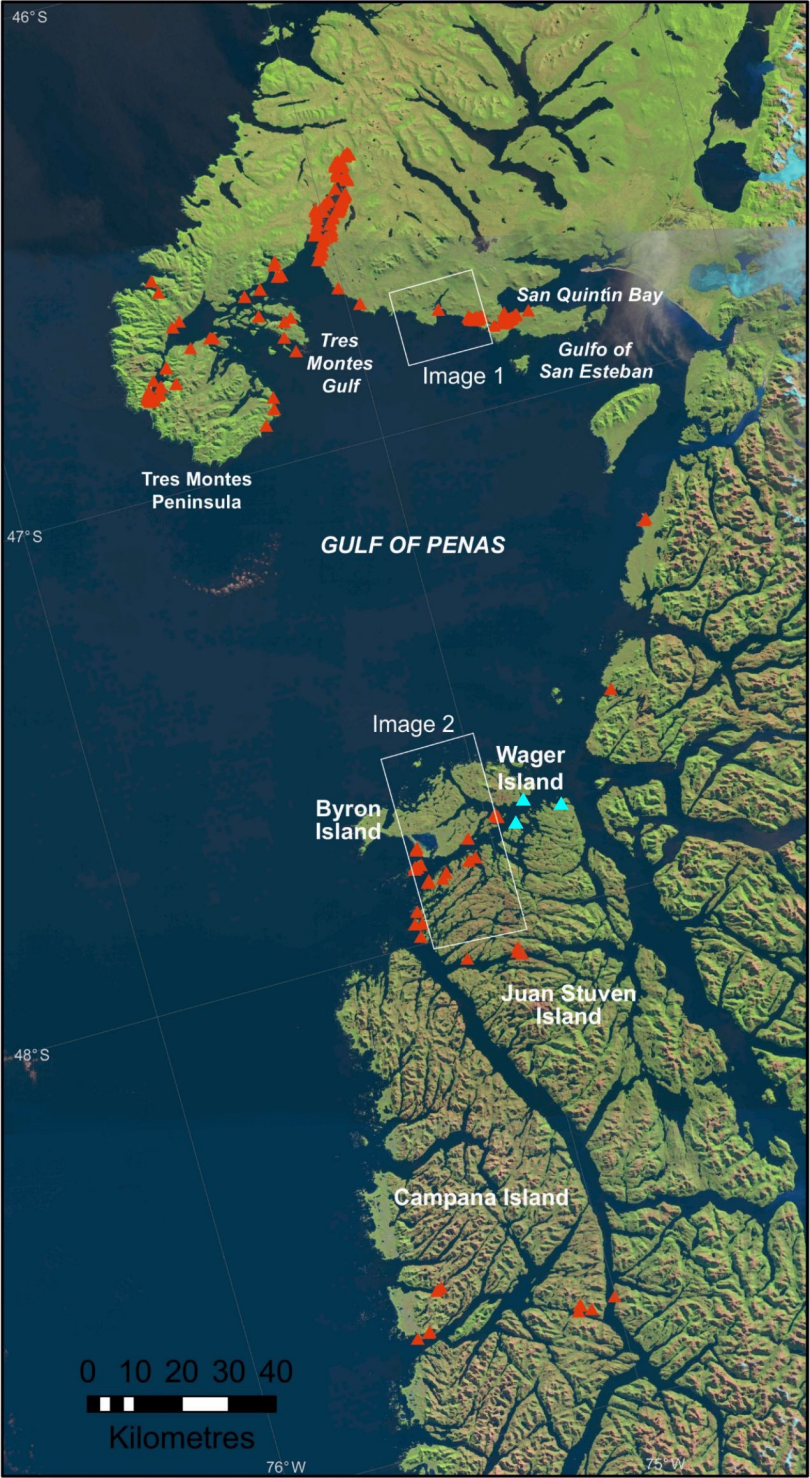
Triangles show the location of whale strandings in the 2015 mass stranding event recorded by boat, aerial or ground survey. The underlying map is a composite of Landsat8 satellite images and shows the complexity of the coastline in the area. The white boxes refer to the extent of the VHR satellite imagery used in this study. The blue triangles show the locations of the three whales identified using WorldView 3 imagery in 2017. Imagery available from USGS Earth Explorer viewer: https://earthexplorer.usgs.gov/.

**Fig 2:**
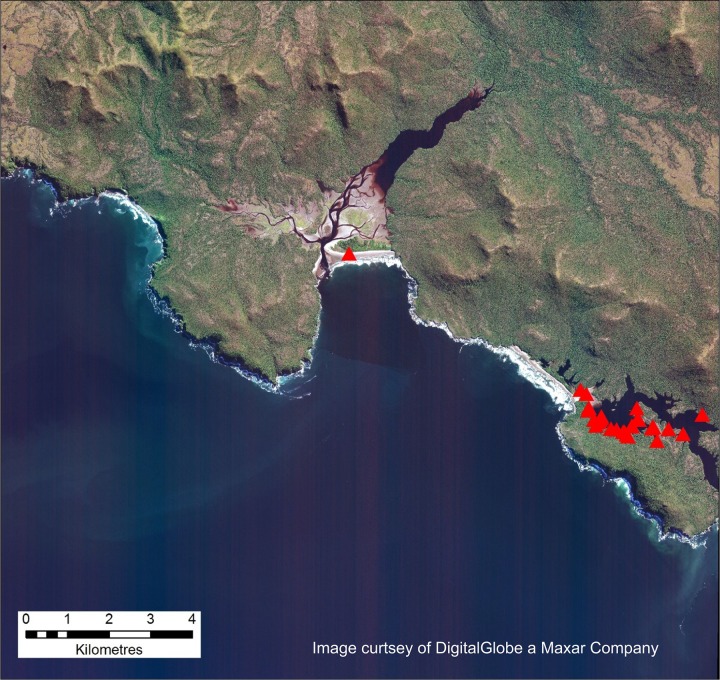
The WorldView2 satellite image of Escondido Sound (Image 1) shown using the visible bands (5/3/2). The red triangles represent the recorded whale strandings from the 2015 mortality event, 31 in total in this area, with 30 in Escondido Sound and 1 on a sandy beach to the center of the image [taken from [25]]. Imagery from DigitalGlobe Products. WorldView2 © 2019 DigitalGlobe, Inc., a Maxar company.

The study area concentrated on two areas around the Gulf of Penas in southern Chile. Here, cloud-free archival VHR satellite imagery was collected between the time that the whales stranded and the period when visual surveys were conducted. The area over which stranded whales were found by boat and aerial survey was extensive and the archival imagery we used only covered a small portion (~15%) of this. The first, smaller area we studied was the western San Quintín Bay (outlined in white on Fig 1). This 16 km x18 km (288 km^2^) area is characterized in the west by Escondido Sound (74°40’W, 46°49’S), a heavily vegetated coastline with a complex network of rills and creeks which link to San Quintín Bay (the main part of which is located further to the east). The remainder of the coast is also vegetated, mainly rocky shoreline, but with two sandy beaches near the outlets of streams and rivers, facing the Gulf of Penas. Thirty-one whales were recorded as stranded from an aerial survey on 24^th^ June 2015 in this stretch of coastline, 30 in Escondido Sound and one on a sandy beach ~7 km to the northwest (Fig 2).

The area of the second satellite image is larger (836 km^2^) and contains several islands on the southern side of the Gulf of Penas, including Wager Island, Byron Island and part of Juan Stuven Island (74°40’W, 47°50’S). The coast of these islands is greatly in-folded with many creeks and fjords, with a mixture of rocky, sandy and vegetated beaches. The density of sei whales stranded in this area was much lower than that in San Quintín Bay, with a total of 15 whales recorded in the aerial survey in the area of the image.

### The satellite imagery

We used two archival WorldView2 images (catalogue ID ID: 103001003F7A9900 date 24^th^ March 2015 and ID: 1030010040C2EE00 date 4^th^ April 2015), comprising of panchromatic (0.5 m spatial resolution) and 8 colour bands (2 m spatial resolution). These were the best available images in the DigitalGlobe archive within the timeframe of the stranding and in areas where stranded whales had been recorded. Image 1, of Escondido Sound, western San Quintín Bay, was taken on 24^th^ March 2015, some three months before the aerial survey, but at least a month after the onset of the mortality event, as estimated by Häussermann *et al*. [25]. Image 2 (catalogue ID 057273808010) was taken ten days later on 4^th^ April 2015 and spanned an area to the south side of the Gulf of Penas, including Byron Island and parts of Wager and Juan Stuven Islands. This larger image contained some cloud in the south-western part. For details on how to search and acquire imagery from the DigitalGlobe Search and Discovery tool (https://discover.digitalglobe.com/) see supplemental data.

The images were radiometrically corrected and pansharpened using the Gram-Schmidt pan sharpening algorithm [28]. Pan-sharpening is an image analysis process that increases the resolution of the multispectral (colour) image pixels, by using the contrast and intensity of the higher resolution panchromatic (grayscale) pixels. Both processes were completed in ENVI software (version 5.4 Harris-Geospatial 2017).

### Manual analysis

The initial analysis consisted of a manual scan and count of whale-like objects on the shoreline in each pansharpened image using ArcGIS software (ArcGIS desktop version 10.4.1 ERSI software 2015). The images were viewed at a scale of 1:1000 and each whale like object identified was recorded by editing a point in the location above the centre of the identified feature. These locations were compared to the locations of the aerial and ground survey in Häussermann et al. [25]. The nearest distance between the satellite and pre-existing GPS location was analyzed using the “Near” tool in ArcGis (Esri®ArcMapTM 10.4.1.5686). Most of the features identified were obvious by their whale-like size and shape (~7 to 20 m in length and roughly oval or cigar shaped) although in some cases, due to their close proximity, the exact number of whale carcasses was uncertain. Each whale-like object was given a confidence ranking: 1) for an obvious whale (right shape and size and fluke visible), 2) for a probable whale (approximately the right shape and size with no possible confounding feature) and 3) for a possible whale (size and shape less whale-like or possible confounding features such as tree trunks). These classes were manually defined by an experienced image analyst (PTF). The color of the decomposing whales was a good additional indicator (see below). Both images 1 and 2 were examined by a single experienced image analyst (PTF). At this resolution, manual analysis took a full day to investigate the first image and two-and-a half days to manually assess the second image.

### Spectral profiles

**Fig 3:**
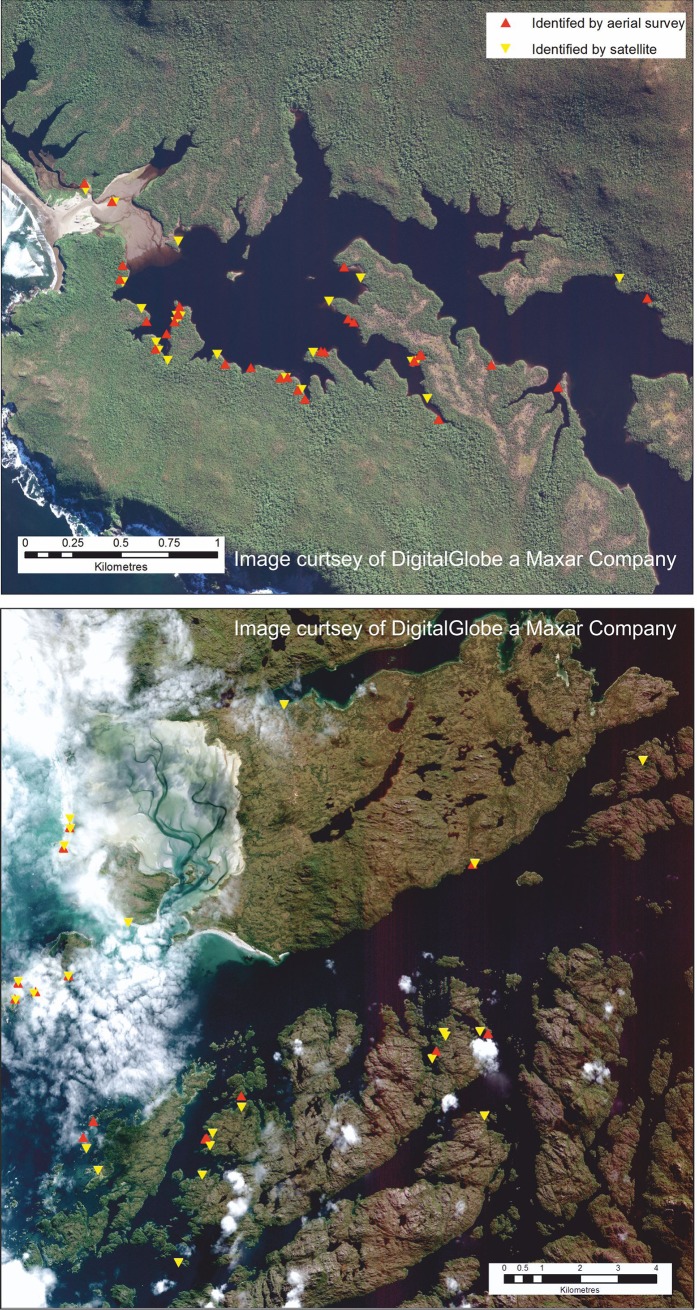
The results of the manual count (yellow triangles) compared to the positions of strandings recorded in the aerial survey (red triangles). The top image (Fig 3A, Image 1) corresponds to Escondido Sound, western San Quintín Bay; the bottom (Fig 3B, Image 2) corresponds to Byron, Wager and Juan Stuven Islands. See Fig 1 for the locations of each area. Imagery from DigitalGlobe Products. WorldView2 © 2019 DigitalGlobe, Inc., a Maxar company.

Photographic evidence from the boat surveys showed that many of the dead whales had partially decomposed and had changed color to a pink or orange hue (in wavelengths visible to the eye). To this end, we collected spectral profiles of specific pixels from the satellite imagery to ascertain if the profiles of these pixels could be used as signatures to identify decomposing whales automatically. Using ENVI software, spectral profiles were taken from nine whales; these comprised of forty pixels from five whales in the northerly image (Image 1, Fig 3A) and twenty pixels from four whales in the southerly image (Image 2, Fig 3B). The pixels were chosen manually from the un-shadowed center portion of each whale-like object, which generally has the brightest pixels with the greatest contrast with the surroundings.

### Automated analysis

Automated analysis consisted of a Spectral Angle Mapper (SAM) [29] procedure conducted in ENVI software combined with a buffer analysis so that only coastal areas were analyzed. The SAM analysis is a target-finding algorithm which attempts to use whale spectral signatures in order to automatically detect other whale like objects in the imagery. The method consists of a two-step process, a number of spectral signatures from target pixels (end members) are identified and input; the analysis then returns pixels that have a similar spectral profile above a user defined threshold. We used pixels from the center of three clearly identified whales as target spectra for the analysis from each image. As the analysis works on the shape of the spectral profile, rather than absolute values, differences in total illumination do not interfere with the spectral results, this allows indices to be built, which are insensitive to sun angle and can be used in different satellite images. The SAM analysis returns pixels above a target likelihood threshold (we gave the threshold of 5%) and returns a “likelihood” value for the pixels that fall within this threshold.

To construct the buffer used to restrict the SAM analysis, an accurate coastline had to be constructed. The available vector coastlines for this area proved too coarse, so we constructed a high-resolution coastline using a Normalized Difference Water Index (NDWI) [30–31] from the atmospherically corrected VHR image. This was buffered so that only pixels within 5m of the coast were included in the main analysis.

In Image 1, where the whale carcasses were concentrated in one portion of the coverage area, two analyses were conducted–the first in a subset of Escondido Sound that contained all the identified whales, and the second of the whole area. In Image 2, as the whales were widely spread, a single analysis of the whole image was conducted.

## Results

### Results of the manual scan

#### Image 1

Twenty-two objects were identified as possible whales from the manual analysis of Image 1, 20 of which were in the creeks of Escondido Sound, the other two being on the flat sandy area just to the west of the San Quintín Bay (see Table 1 for summary). The single stranding recorded by Häussermann et al. [25] on the sandy beach to the North West of San Quintín could not be found. Most of the objects identified as possible whales were between 9–15 m in length and had a clear whale-like shape. Most of them also had an orange to pink hue in the pan-sharpened imagery, which was also reflected in photos of decomposing carcasses from the ground and aerial surveys. Identified objects were grouped into three classes (see Methods section for details). Of the 22 objects, 11 were counted as class 1 (obvious, high confidence), 3 as class 2 (probable) and 8 as class 3 (possible). Most of the suspected whale carcasses in class 3 were adjacent or overlapping to more obvious whale-like features, making the estimation of numbers more difficult, and it is possible that an underestimate of numbers occurred because of this.

**Table 1:** Number of whales found in the satellite survey compared to ground survey (from boat and air) in the same areas (see text for explanation of class ranking).

	Ground survey	satellite survey	class 1	class 2	class 3
Image 1	30	22	11	2	8
Image 2	14	23	14	3	6

The greatest distance of a whale-like feature on the image from an aerially surveyed whale was 315 m and all of the remaining whales located by satellite were within 200 m of a whale identified from the aerial survey, whilst 79% (19 of 24) were within 100 m (S1 Table). No whales were identified in the imagery along other parts of the coastline.

#### Image 2

The number of whales counted in the second image, of Byron and Wager Island, was considerably more than in the aerial survey of the same area. Twenty-three potential whale carcasses were manually counted in the satellite image, 14 with high confidence (class 1), three with moderate confidence (class 2) and six with low confidence (class 3). Overall, the objects on Image 2 were more clearly whale-like than on Image 1, probably due to the lack of crowding evident in Escondido Sound. Of the 23, seven were within 100m of the position of a whale recorded in the aerial survey, whilst a further six were within 300 m, and two others were within a kilometer. If it is assumed that the remaining 14 whales recorded in the aerial survey correspond to the nearest whale in the satellite image, this leaves nine extra whale-like objects on the satellite image that were not recorded in the aerial survey. Of these, three had a low confidence ranking (class 3), whilst five had a high (class 1) ranking and one a moderate (class 2). In areas of thin cloud, whale-like features could generally still be detected with some confidence.

### Spectral profiles

**Fig 4:**
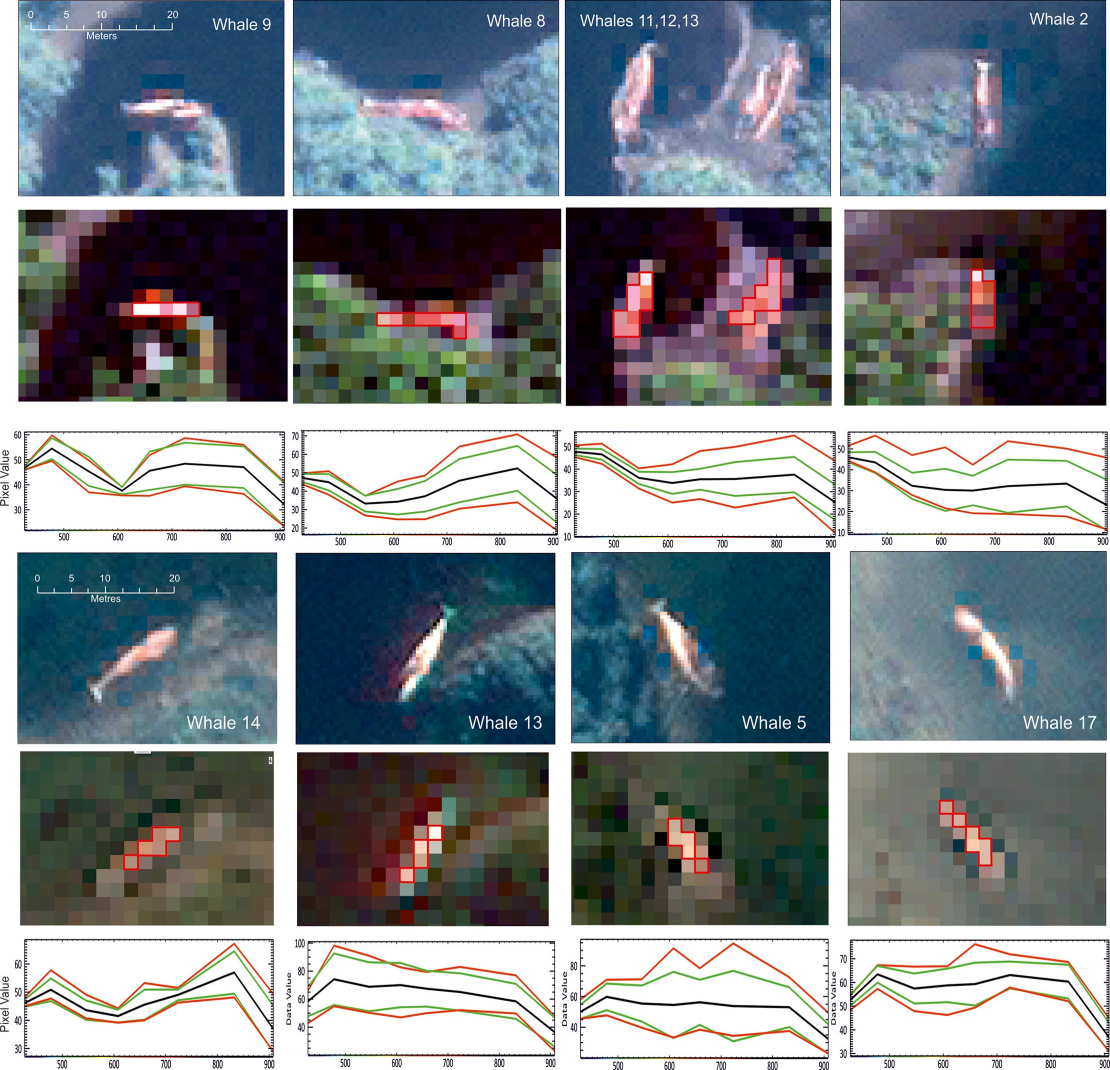
Image chips and spectral profiles from ten whales. At the top are whales from Image 1 and the bottom are from Image 2. The upper row displays detailed pan-sharpened images (spatial resolution 0.5 m) shown using visible bands of the satellite imagery. The second row is the multispectral band (2 m resolution pixels) used in the spectral analysis with the area of pixels chosen for each profile highlighted in red. The third row are the spectral profiles for each image showing Top-Of-Atmosphere radiance in the Y axis and wavelength in nanometers in the X axis. Imagery from DigitalGlobe Products. WorldView2 © 2019 DigitalGlobe, Inc., a Maxar company.

The analysis of spectral profiles was carried out for both the multispectral and pan-sharpened images (Fig 4). There are some differences between the four spectral profiles from Image 1 and Image 2. The mean (black line) profiles of whales in Image 1 are more congruent with each other than those in Image 2. All those in Image 1 have a slight peak at 850 nm (band 7 of the imagery). The spectra of whales 11–13 (Fig 4) and whale 2 are very similar, with a generally flat profile from 550–850 nm (bands 3–7). However, the min-max and standard deviation of the profiles in Image 1 are generally quite wide (Fig 4) suggesting that there is a large amount of spectral heterogeneity within each whale-like object. Image 2 shows more variety in the profiles, although there is some agreement between the mean results in the whale 13 and whale 5 profiles. Many of the pixels in the second image are generally brighter, with radiance values of over 60, possibly suggestive of differing lighting conditions. Interestingly, the high reflectance in the red and NIR bands can be used as a factor to discriminate stranded whales from some possible confounding features such as logs and waves, which, although having similar shape do not have similar spectral properties.

### Spectral angle mapper

#### Image 1

The results from the SAM analysis are very different for Image 1 and Image 2. In Image 1, the analysis confined to the area around the strandings returned 39 pixels that were most likely to be purely whale; 26 of these pixels (66.7%) were associated with the whale carcasses identified by the manual search of the image. The SAM pixels were clustered into 24 groups, identifying 14 of the 21 (66.7%) manually identified whale carcasses (half of the whales were identified by groups of pixels rather than single pixels). The SAM analysis returns a “likelihood value” and the sixteen pixels deemed most likely to be whales, comprising all the likelihood values above 40, were all related to whale carcasses. There were a number of errors of commission (i.e objects identified as whales that are not whales) (see Table 2), these were mainly on sandy beaches or coastal mud flats with a similar spectral signature; all of these had lower likelihood values. There were 7 out of 14 whales not identified by the analysis (errors of omission).

**Table 2:** Results of the two automated Spectral Angle Mapper analyses from image 1, showing the pixels identified as “whale” in the automated procedure and the associated errors. Spectral Angle mapper returns pixels with associated “likelihood” results, these values are associated with how closely (between 1–100) each identified pixel matches the training data. By thresholding these values, the algorithm can be tuned to remove errors of omission or errors of commission. Here we give pixel results with three “Likelihoods”; all likelihoods, likelihoods over 40 and likelihoods over 65.

	Escondido Sound		Total Image	
	No. pixels	% of pixels	No. pixels	% of pixels
**All likelihoods**				
Total identified pixels	39	100.0	109	100
whale pixels	26	66.7	26	23.9
errors of commission	13	33.3	83	76.1
whales identified	14 of 21	66.7	14 of 23	60.8
errors of omission	7	33.3	9	39.2
**Likelihood value > 40**				
pixels	16	41.0	28	25.6
whale pixels	16	100.0	16	57.1
errors of commission	0	0.0	12	42.9
whales identified	11	52.3	11	47.8
errors of omission	10	47.7	12	52.2
**Likelihood value > 65**				
pixels	10	25.6	12	11
whale pixels	10	100.0	10	83.3
errors of commission	0	0.0	2	16.7
whales identified	6	28.6	6	26.1
errors of omission	15	71.4	17	73.9

When analyzing the whole of Image 1, more errors of commission were found; there were 70 extra pixels identified outside of Escondido Sound that were not associated with whales. Most of these pixels had low likelihood values; only 12 pixels that had a likelihood of over 40 were not associated with whales and this reduced to two (out of 12) pixels that had a likelihood over 65.

#### Image 2

The results from analysis of Image 2 were generally poorer. As several of the strandings occurred on beaches above the tide line, a buffer of 5 m from the coast (constructed in Image 1 using NWDI) was inappropriate. In addition, some of the image contained cloud, which was a confounding feature in the SAM analysis. Cloud in this image had to be highlighted as an extra signature and removed by the SAM analysis. After a number of attempts, the best SAM analysis could only identify 5 of the 23 possible whales, with several hundred erroneous pixels as errors of commission. Most of these errors were in cloud or in forested areas however, many were at the coastal fringe, within a few meters of the shoreline. As this area was where many of the whales were located, automatically differentiating whales from non-whale features on this image was not effective.

## Discussion

Opportunistic collection of satellite images close in time to the sei whale stranding event revealed that the numbers of stranded whales may have been higher than was estimated by traditional survey methods three months after the first report of the event. However ascertaining the exact amount is difficult, as neither the aerial nor satellite survey have full or systematic coverage of the area.

The analyses of satellite imagery collected after the sei whale stranding event in the Gulf of Penas confirm how satellite images can be used to efficiently measure stranded whale numbers and distribution, providing an assessment of a stranding and its impact more rapidly and at lower cost than traditional surveys and without being limited by geographical remoteness. Spectral analysis of these images showed that part of the spectral profile, in the red and NIR, can also be used to differentiate stranded whales from some of the possible confounding features such as logs or waves, as these both have relatively low reflectivity in bands 5–8 of this imagery. In some circumstances, use of these spectral profiles may provide better object discrimination than aerial surveys.

### Manual counts

The close match between the aerial survey locations and whales identified in the satellite imagery shows that VHR satellite images can be used to identify and count large stranded baleen whales. Due to the three months temporal difference in the respective surveys, it is not possible to test the accuracy of this approach relative to aerial survey. However, all except one of the whales identified in Image 1 were within 200m of a carcass identified from the aerial survey, and whales were found near all of the aerial survey points for Image 2, which suggests good concordance between the images and surveys. The close agreement of counts and carcass locations from Image 1 and the aerial survey shows that by the 24th of March 2015 most of the mortality in Escondido Sound had already happened, although, as some carcasses were in close contact or obscuring each other, the exact number of strandings was difficult to ascertain by satellite.

On Image 2, nine more whales were identified from the satellite image than from the aerial survey (an extra 64%), of which five were of class 1 or 2. Three of these were in areas not covered by the aerial survey, but four were in areas near to the track of the plane and, we assume, would have been spotted if they were there at the time of the fly-over. As the aerial survey took place later than the satellite image it is possible that many carcasses had washed out to sea and sunk. As hypothesized in Häussermann et al. [25] it seems quite likely that the 343 whales recorded in this, the largest ever baleen whale stranding event, were an underestimate.

The locations of the objects identified by satellite images matched roughly with those from the later aerial survey, although the exact locations differed. This can be explained by three factors: (1) during the aerial survey there were logistic restrictions for recording GPS coordinates of the whale carcasses, so coordinates were marked using the plane route and a posteriori corrections made using geomorphological landmarks; (2) carcass movements due to high tides; (3) detection error during the aerial survey. Generally, the difference in the matches between the aerial and satellite image locations in image 2 (southern Gulf of Penas, including Wager and Byron Islands) was greater than on image 1 (western San Quintín Bay, including Escondido Sound). During the aerial survey, the survey over Escondido Sound was repeated four times due to the highest density of the whales in that place, while it was only repeated twice in the vicinity of Byron Island (field observation CSG and VH). However, a bigger factor explaining the difference may be that western San Quintín Bay (image 1) is a sheltered location with little ocean wave energy, but the southern Gulf of Penas area (image 2) corresponds to more exposed geomorphological and oceanographic conditions of the coast especially on the western side and has some of the most energetic tides in the whole gulf [Häussermann et al. 2017].

### Automated analysis

The long, complex and indented coastline of southern Chile would be time consuming and difficult to examine manually at the resolution required to identify whale strandings in satellite imagery. We therefore explored automated and semi-automated procedures, to see if they could help with the process of finding stranded whales in the satellite images. Our initial analysis was based on a simple spectral target-finding algorithm, relying purely on the differences in the spectral profile of the beached whales. While the pixel-based analysis could identify whales, there were many errors of commission and omission. SAM analysis identified 61% of the manually identified whales in Image 1 but only 22% of those manually identified in Image 2. These results reflect the substantial variation in pixels seen within the spectral profiles of whales in both images. This is probably due to the heterogeneous spectral reflectivity of each whale carcass due to different states of decay; carcass images shown in Häussermann et al. [25] show a large variation in visible colour depending upon the state of decay. The decay is mostly of the skin tissue which made the overall coloration of the whales on the image very varied. Ground and aerial surveys reported that many whales were beached ventral side up, so some were dark or light depending upon their orientation. This problem was exacerbated depending on whether the carcass was beached or partially floating, in sun or shade, or how the carcass was orientated in relation to the sun. Image 2 presented additional problems including partial cloud cover over some of the whales, shadowed whales and whales stranded on the upper part of beaches way away from the water, which rendered the results from the SAM analysis very poor.

It may be possible to improve the chances of detection and reduce the number of false positives by using more complex Rule-Based [e.g. [32]] or Object-Based Image Analysis routines [e.g. [33]], but due to the variety of shapes and heterogeneous spectra it is unlikely that a totally automated solution can be constructed using the current resolution imagery. A semi-automated solution, where automation highlights the most likely areas and manual counts are used on these areas is much more likely to provide a solution. However, with the recent availability of higher resolution satellite sensors, such as WorldView3 and WorldView4, with 31 cm rather than 50 cm spatial resolution, the prospect for automating this process may improve due to better shape discrimination and the larger number of pixels available for identification of each whale.

### Implications for cetacean conservation and future directions

Here we demonstrate how the strategic collection of satellite imagery has potential to provide a quick and relatively reliable view of a whale stranding event. Limitations in using satellite imagery in comparison to traditional surveys are:

1. Discrimination of individuals can be difficult when whales are tightly grouped (i.e. individuals may be under-counted)
2. Automation of the approach is not yet reliable and manual searching of images can be time-consuming; whales cannot be directly identified to species.
3. Cloudy weather conditions when the image is collected can reduce resolution.
4. Satellite imagery cannot be tasked immediately (e.g. a time window of at least a week has to be given with the request) which would limit urgent response to a stranding notification, nevertheless in comparison to the current time of response of several weeks-months at the studied area, for example, it represents a great improvement. In this sense, it could be a powerful tool for monitoring events in remote areas, such as certain parts of Chilean Patagonia, where no regular visits are regularly implemented due to extreme weather conditions and elevated cost.

(For discussion on how to access VHR satellite imagery and the cost of imagery see S1 Text and S2 Text).

For more a more general critical assessment of the use of satellite imagery when counting larger animals see [34–36].

With respect to discerning group sizes of whales, this uncertainty might create a negative bias in absolute numbers of stranded whales, but it is usually possible to discern when more than one whale is present, even if absolute numbers in that group are harder to assess. The future use of newly available higher resolution imagery (i.e. Worldview 3 and 4, 31cm resolution) may also reduce this problem as it has greater discrimination power. With respect to image search times, manual searching has proven to be the most reliable means of identifying whales at present, but with careful planning a combination of manual and semi-automated searching is likely to reduce image searching times and make large-scale satellite imagery interrogation more feasible. Identification of whales to species would require investigation following indication of a stranding event using satellite imagery. In some situations, it may be possible to provide tentative diagnosis based on size and general morphology, but where carcasses are involved and whales are often ventral-side up, direct inspection is usually necessary [25].

**Fig 5:**
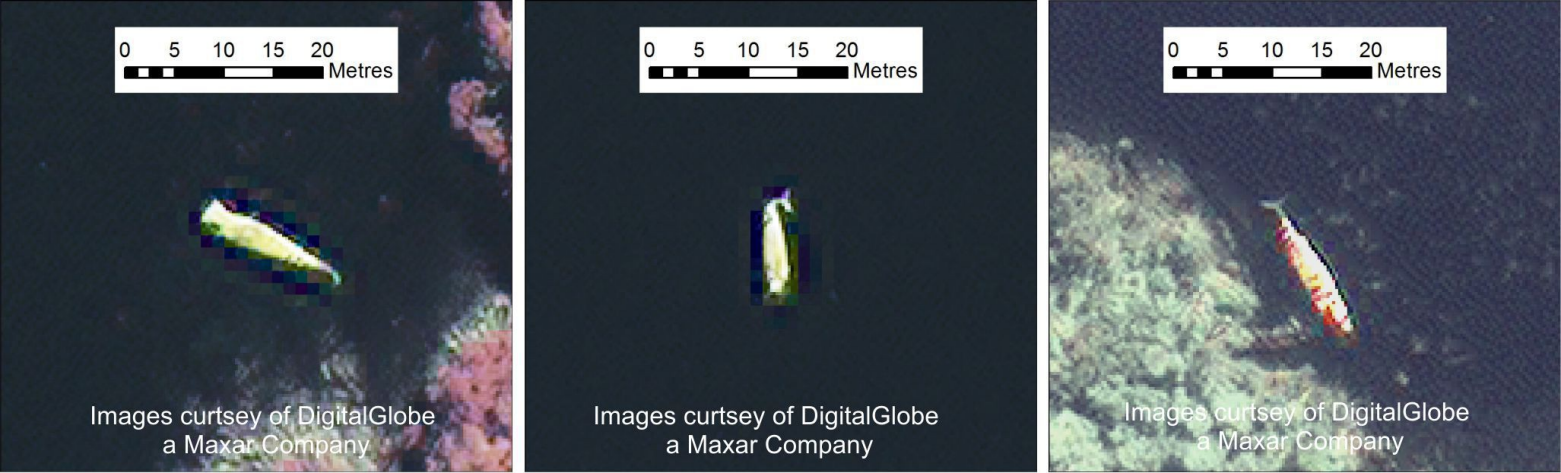
Examples of stranded whales in WorldView3 imagery from the Gulf of Penas in March 2017, showing the improved shape discrimination using this higher resolution data. The locations of these whales are shown as blue triangles in Fig 1. Imagery from DigitalGlobe Products. WorldView3 © 2019 DigitalGlobe, Inc., a Maxar company.

The minimum size at which animals can be discerned is not yet understood, and is likely to vary depending on the resolution of the image collected, the size of the animal, contrast with its surrounding environment and possible confounding features. The highest current resolution 31 cm imagery was not available at the time of the 2015 Gulf of Penas mass stranding event, but collection of a small amount of this imagery in 2017 from the same area confirms that whales are much easier to identify with this higher resolution data (see Fig 5).

Further work, using high-resolution images to establish the capacity of the technique to discern cetaceans of different sizes is important. This will help us to understand if, for example, calf strandings are missed, and the capacity of the approach to monitor strandings of smaller cetaceans. A systematic search is needed to determine which archival imagery may exist for other historical events and may be informative on these questions. Further work is also required to establish the sizes of animals that can be discerned, by obtaining satellite images of known-size stranded cetaceans of differing sizes. This is also important for analyzing strandings of odontocetes, which occur much more regularly than mass strandings of baleen whales. One potentially very useful application of this technique is to provide regular snapshots of remote areas where whale strandings are relatively likely, in order to monitor stranding patterns and ‘catch’ events close to the time that they happen. South American Patagonia, Tasmania, New Zealand and the Falkland Islands (Islas Malvinas) have large areas of remote coastline and regularly experience cetacean group strandings; these may be ideal candidates for such monitoring.

## Conclusion: Expanding the analysis and future directions

This study confirms the 2015 MME in the Gulf of Penas, and, by a quantifiable comparison with aerial and boat survey data, shows that large whales can be reliably detected and counted in VHR satellite imagery. The ease of access to satellite imagery, low potential cost, large spatial coverage and relatively simple logistics means that VHR satellite imagery could become a valuable tool for monitoring MME and other strandings events in remote areas.

Our paper also suggests that the 343 whales counted in the Gulf of Penas mass mortality event is an underestimate. Analysis of other VHR imagery from the satellite archive could help further quantify the magnitude of the underestimate and the full extent of the stranding area. More work is needed to construct an automated or semi-automated procedure to count stranded whales. Future work should focus on the investigation of machine learning algorithms, including rule based and object based automated analysis with the use of higher resolution (31cm) satellite imagery to improve capacity to search the imagery. Overall, satellite imagery may be a cost effective and safer alternative to aerial survey for future assessment of the extent of mass whale stranding events.

## Supporting information

S1 Table Data tables: Table of locations of stranded whales found in the two satellite images. Near distance relates to how close, in metres, each whale was to the nearest corresponding whale in Hausserman et al. 2018. See this publication for a full list of the whales found on the aerial and boat survey. For confidence see main text: 1 = high, 2 = moderate, 3 = low.
(DOCX)

S1 Text Ordering of high resolution satellite imagery. (DOCX)

S2 Text Cost of satellite imagery. (DOCX)

## References

[1] Saavedra C, Pierce GJ, Gago J, Jusufovski D, Cabrero A, Cervino S, et al (2017) Factors driving patterns and trends in strandings of small cetaceans. *Mar Biol*. 164 (8). ARTN 165 10.1007/s00227-017-3200-3

[2] Perrin W.F., Geraci J.R. (2008) Stranding In: *Encyclopedia of Marine Mammals* (eds. Perrin WF, Würsig B, Thewissen JGM), pp. 1118–1123. Academic Press, New York.

[3] Rowntree V.J., Uhart M.M., Sironi M., Chirife A., Di Martino M., La Sala L., et al (2013) Unexplained recurring high mortality of southern right whale Eubalaena australis calves at Península Valdés, Argentina. *Mar*. *Ecol*. *Prog*. *Ser*. 493, 275–289.

[4] Evans K., Hindell M., Hince G. (2004) Concentrations of organochlorines in sperm whales (Physeter macrocephalus) from Southern Australian waters. *Mar*. *Pollut*. *Bull*. 48, 486–503. 10.1016/j.marpolbul.2003.08.026 14980465

[5] Evans K., Thresher R., Warneke R.M., Bradshaw C.J.A., Pook M., Thiele D., et al (2005) Periodic variability in cetacean strandings: links to large-scale climate events. *Biol*. *Lett*. 1. 10.1098/rsbl.2005.0313 PMC1626231 17148151

[6] Rosas CL, Gil MN, Uhart MM. (2012) Trace metal concentrations in Southern Right Whale (Eubalaena australis) at Peninsula Valdes, Argentina. *Mar Pollut Bull*. 64 (6):1255–60. 10.1016/j.marpolbul.2012.02.026 22465055

[7] Lair S, Measures LN, Martineau D. (2016) Pathologic Findings and Trends in Mortality in the Beluga (Delphinapterus leucas) Population of the St Lawrence Estuary, Quebec, Canada, From 1983 to 2012. *Vet Pathol*. 53 (1):22–36. 10.1177/0300985815604726 26374277

[8] Truchon M.H., Measures L., L'Herault V., Brethes J.C., Galbraith P.S., Harvey M., et al (2013) Marine Mammal Strandings and Environmental Changes: A 15-Year Study in the St. Lawrence Ecosystem. Plos One 8. 10.1371/journal.pone.0059311 PMC3609766 23544059

[9] Pyenson N.D., Gutstein C.S., Parham J.F., Le Roux J.P., Carreño Chavarría C., Little H., et al (2014) Repeated mass strandings of Miocene marine mammals from Atacama Region of Chile point to sudden death at sea. P Roy Soc B-Biol Sci 281. 10.1098/rspb.2013.3316 PMC3953850 24573855

[10] Nash S.M.B., Baddock M.C., Takahashi E., Dawson A., Cropp R. (2017) Domoic acid poisoning as a possible cause of seasonal cetacean mass stranding events in Tasmania, Australia. *Bull*. *Environ*. *Contam*. *Toxicol*. 98, 8–13. 10.1007/s00128-016-1906-4 27530123

[11] Oremus M., Gale R., Kettles H., Scott Baker C. (2013) Genetic Evidence of Multiple Matrilines and Spatial Disruption of Kinship Bonds in Mass Strandings of Long-finned Pilot Whales, Globicephala melas, Journal of Heredity, Volume 104, Issue 3, Pages 301–311, 10.1093/jhered/est007 23493607

[12] Mannino M.A., Talamo S., Tagliacozzo A., Fiore I., Nehlich O., Piperno M., et al (2015) Climate-driven environmental changes around 8,200 years ago favoured increases in cetacean strandings and Mediterranean hunter-gatherers exploited them. Sci Rep-Uk 5. 10.1038/srep16288 PMC4648091 26573384

[13] Dalebout M. L., Van Helden A., Van Waerebeek K., Baker C. S. (1998), Molecular genetic identification of southern hemisphere beaked whales (Cetacea: Ziphiidae). Molecular Ecology, 7: 687–694. 10.1046/j.1365-294x.1998.00380.x 9640649

[14] Pyenson N.D. (2011) The high fidelity of the cetacean stranding record: insights into measuring diversity by integrating taphonomy and macroecology. *P Roy Soc B-Biol Sci* 278, 3608–3616. 10.1098/rspb.2011.0441 PMC3189373 21525057

[15] Peltier H., Dabin W., Daniel P., Van Canneyt O., Doremus G., Huon M., Ridoux V. (2012) The significance of stranding data as indicators of cetacean populations at sea: Modelling the drift of cetacean carcasses. *Ecol*. *Indicators* 18, 278–290.

[16] Sundaram B., Poje A.C., Veit R.R., Nganguia H. (2006) Acoustical dead zones and the spatial aggregation of whale strandings. *J*. *Theor*. *Biol*. 238, 764–770. 10.1016/j.jtbi.2005.06.022 16083913

[17] Coughran D.K., Gales N.J., Smith H.C. (2013) A note on the spike in recorded mortality of humpback whales (Megaptera novaeangliae) in Western Australia. *J*. *Cetacean Res*. *Manage*. 13, 105–108.

[18] Jepson P.D., Arbelo M., Deaville R., Patterson I.A.P. (2003) Gas-bubble lesions in stranded cetaceans—Was sonar responsible for a spate of whale deaths after an Atlantic military exercise? *Nature* 425, 575–576. 10.1038/425575a 14534575

[19] Jepson P.D., Deaville R., Patterson I.A.P., Pocknell A.M., Ross H. M., Baker J.R., et al. (2005) Acute and chronic gas bubble lesions in cetaceans stranded in the United Kingdom. *Vet*. *Pathol*. 42, 291–305. 10.1354/vp.42-3-291 15872375

[20] Peltier H, Ridoux V. (2015) Marine megavertebrates adrift: A framework for the interpretation of stranding data in perspective of the European Marine Strategy Framework Directive and other regional agreements. *Environ*. *Sci*. *Policy*. 54, 240–7. 10.1016/j.envsci.2015.07.013

[21] Fretwell P.T., Staniland I.J. & Forcada J. (2014) Whales from space: counting southern right whales by satellite. PloS one, 9 (2) e88655 10.1371/journal.pone.0088655 24533131 PMC3922973

[22] Cubaynes H.C., Fretwell P.T., Bamford C., Gerrish L. & Jackson J.A. (2018) Whales from space: Four mysticete species described using new VHR satellite imagery Marine Mammal Science, 2018, 10.1111/mms.12544

[23] Geraci JR, Anderson DM, Timperi RJ, Staubin DJ, Early GA, Prescott JH, et al (1989) Humpback whales (Megaptera novaeangliae) fatally poisoned by dinoflagellate toxin. *Can*. *J*. *Fish Aquat*. *Sci*. 46 (11):1895–8. 10.1139/f89-238

[24] Hucke-Gaete R, Viddi F, Cassis D, Bedriñana L, Häussermann V, Pérez-Alvarez MJ., et al. eds. Informe técnico sobre la mortalidad masiva de ballenas en Puerto Slight y Caleta Buena, Golfo de Penas, Región de Aysén (expedición de mayo 2015). In: Puerto Aysen: Fiscalía de Aysen. Fiscalía de Aysén. 2015. Official request SIAC nr 460428815 for report.

[25] Häussermann V., Gutstein C.S., Beddington M., Cassis D., Olavarria C., Dale A.C., et al (2017) Largest baleen whale mass mortality during strong El Niño event is likely related to harmful toxic algal bloom. PeerJ Preprints 10.7287/peerj.preprints.2707v1. PMC6055221 30038848

[26] Ulloa,. M.A.E., (2015) Gulf of Penas expedition, Mysticetes Large Mortality Event in Chile. Sernapesca internal report. Ministerio de Economai, Fomento y Turismo, Chile.

[27] SERNAPESCA (2016) SERNAPESCA statistical report 2016, http://www.sernapesca.cl/informes/estadisticas

[28] Maurer T. (2013), How to pan-sharpen images using the gram-schmidt pan-sharpen method–A recipe. International Archives of the Photogrammetry, Remote Sensing and Spatial Information Services, vol xl-1/w2, ISPRS, Hannover Workshop 2013.

[29] Kruse F. A., Lefkoff A. B., Boardman J. B.,. Heidebrecht K. B, Shapiro A. T., Barloon P. J., et al "The Spectral Image Processing System (SIPS)—Interactive Visualization and Analysis of Imaging spectrometer Data." Remote Sensing of Environment 44 (1993): 145–163.

[30] McFeeters S.K. (2007) The use of the Normalized Difference Water Index (NDWI) in the delineation of open water features. Remote Sensing Letters, Pages 1425–1432, 10.1080/01431169608948714

[31] Gao B.-C. 1996 NDWI -A normalized difference water index for remote sensing of vegetation liquid water from space. Remote Sensing of Environment 58: 257–266 10.1117/12.210877

[32] Fretwell P.T., Scofield P., Phillips R.A., (2017) Using super-high resolution satellite imagery to census threatened albatrosses. Ibis 159, (3) 481–490.

[33] Blaschke T. (2009) Object based image analysis for remote sensing. ISPRS Journal of Photogrametry and Remote Sensing. 65 (1) 2–16. 10.1016/j.isprsjprs.2009.06.004 PMC3945831 24623958

[34] Larue M.A. & Knight J. (2014) Applications of very high-resolution imagery in the study and conservation of large predators in the Southern Ocean. *Conservation Biology*, 28, 1731–5. 10.1111/cobi.12367 25103277

[35] LaRue M.A., Stapleton S., Anderson M. (2016), Feasibility of using high-resolution satellite imageryto assess vertebrate wildlife populations. *Conservation Biology*, 31, 213–220 10.1111/cobi.12809 27564920

[36] Hollings T, Burgman M, Andel M, Gilbert M, Robinson T, Robinson A. (2018) How do you find the green sheep? A critical review of the use of remotely sensed imagery to detect and count animals. Methods Ecol Evol., 9: 881–892. 10.1111/2041-210X.12973

