# Correction: Using remote sensing to detect whale strandings in remote areas: The case of sei whales mass mortality in Chilean Patagonia

**DOI:** 10.1371/journal.pone.0225209

**Published:** 2019-11-07

**Authors:** Peter T. Fretwell, Jennifer A. Jackson, Mauricio J. Ulloa Encina, Vreni Häussermann, Maria J. Perez Alvarez, Carlos Olavarría, Carolina S. Gutstein

In the funding statement, the description of funding awarded to Maria Jose Perez-Alvarez is incomplete. The correct information is as follows: Maria Jose Perez-Alvarez was funded by CONICYT-FONDECYT Initiation Program 11170182, Postdoctoral FONDECYT Program 3140513, Projects ICM P05-002 and PFB 023.

## References

[1] Fretwell PT, Jackson JA, Ulloa Encina MJ, Häussermann V, Perez Alvarez MJ, Olavarría C, et al (2019) Using remote sensing to detect whale strandings in remote areas: The case of sei whales mass mortality in Chilean Patagonia. PLoS ONE 14(10): e0222498 10.1371/journal.pone.0222498 31622348 PMC6797088

