# Supplementary material for: Using remote sensing to detect whale strandings in remote areas: The case of sei whales mass mortality in Chilean Patagonia
Source: PLoS One. 2019 Oct 17;14(10):e0222498. doi: 10.1371/journal.pone.0222498 (PMC6797088; doi:10.1371/journal.pone.0222498)
Supplement: S1 Table — Near distance relates to how close, in metres, each whale was to the nearest corresponding whale in Hausserman et al. 2018. See this publication for a full list of the whales found on the aerial and boat survey. For confidence see main text: 1 = high, 2 = moderate, 3 = low. (DOCX) [file pone.0222498.s001.docx]

S1 Data tables

Table of locations of stranded whales found in the two satellite images. Near distance relates to how close, in metres, each whale was to the nearest corresponding whale in Hausserman et al 2018. See this publication for a full list of the whales found on the aerial and boat survey.

For confidence see main text: 1= high, 2=moderate, 3= low.

| Image 1 |  |  |  |  |
| --- | --- | --- | --- | --- |
| ID | NEAR_DIST | confidence | latitude | longitude |
| 0 | 22.53917 | 3 | -46.8153 | -74.6923 |
| 1 | 20.09083 | 1 | -46.8153 | -74.6923 |
| 2 | 315.0406 | 1 | -46.8134 | -74.6885 |
| 3 | 176.5391 | 2 | -46.815 | -74.6585 |
| 4 | 100.7937 | 1 | -46.8151 | -74.6761 |
| 5 | 140.2739 | 3 | -46.8161 | -74.6782 |
| 6 | 8.301327 | 1 | -46.8189 | -74.6722 |
| 7 | 125.8563 | 3 | -46.8207 | -74.6715 |
| 8 | 49.11416 | 1 | -46.8185 | -74.6793 |
| 9 | 19.01757 | 1 | -46.8203 | -74.6801 |
| 10 | 12.42069 | 1 | -46.8197 | -74.6812 |
| 11 | 9.920008 | 1 | -46.8168 | -74.6886 |
| 12 | 13.62685 | 1 | -46.8169 | -74.6884 |
| 13 | 10.7915 | 1 | -46.8169 | -74.6884 |
| 14 | 1.623102 | 3 | -46.8171 | -74.6887 |
| 15 | 18.28459 | 2 | -46.8185 | -74.6899 |
| 16 | 34.28932 | 1 | -46.8181 | -74.69 |
| 17 | 88.0516 | 3 | -46.8189 | -74.6892 |
| 18 | 12.08964 | 3 | -46.8115 | -74.6929 |
| 19 | 35.88473 | 3 | -46.811 | -74.6949 |
| 20 | 19.12823 | 3 | -46.8189 | -74.6722 |
| 21 | 17.53267 | 2 | -46.8198 | -74.6813 |
|  |  |  |  |  |
| Image 2 |  |  |  |  |
| ID | NEAR_DIST | confidence | latitude | longitude |
| 0 | 57.99587 | 1 | -47.8187 | -75.0608 |
| 1 | 57.25704 | 1 | -47.8143 | -75.205 |
| 2 | 33.06029 | 1 | -47.8102 | -75.2029 |
| 3 | 222.9651 | 1 | -47.808 | -75.203 |
| 4 | *298.8119 | 3 | -47.8762 | -75.143 |
| 5 | *962.2159 | 1 | -47.8923 | -75.1569 |
| 6 | *216.6038 | 1 | -47.8853 | -75.1541 |
| 7 | *308.3292 | 2 | -47.8859 | -75.1975 |
| 8 | *951.2211 | 2 | -47.8911 | -75.1934 |
| 9 | *3331.825 | 3 | -47.9129 | -75.1653 |
| 10 | *2133.113 | 3 | -47.8783 | -75.0575 |
| 11 | 233.261 | 3 | -47.8648 | -75.0759 |
| 12 | *516.0823 | 1 | -47.8586 | -75.0719 |
| 13 | *220.4334 | 1 | -47.8584 | -75.0591 |
| 14 | *6482.386 | 1 | -47.7811 | -75.1278 |
| 15 | *459.7526 | 2 | -47.8594 | -75.0712 |
| 16 | *5228.373 | 3 | -47.7944 | -75.0019 |
| 17 | 34.69656 | 1 | -47.8507 | -75.2222 |
| 18 | 72.44047 | 3 | -47.8492 | -75.2159 |
| 19 | 26.75611 | 1 | -47.8464 | -75.2213 |
| 20 | 11.10484 | 1 | -47.8451 | -75.2037 |
| 21 | *2094.194 | 1 | -47.8326 | -75.1826 |
| 22 | 193.7495 | 1 | -47.8824 | -75.1532 |

*whales seen on imagery >200 m distant from sightings survey location.
