# Supplementary material for: Using remote sensing to detect whale strandings in remote areas: The case of sei whales mass mortality in Chilean Patagonia
Source: PLoS One. 2019 Oct 17;14(10):e0222498. doi: 10.1371/journal.pone.0222498 (PMC6797088; doi:10.1371/journal.pone.0222498)
Supplement: S1 Text — (DOCX) [file pone.0222498.s002.docx]

S1 Text: Ordering of high resolution satellite imagery

Very high resolution imagery is available from a number of commercial companies. We have used imagery from the DigitalGlobe Corporation, which owns a constellation of VHR satellites. Image 1 and image 2 were from the WorldView 2 satellite. These were acquired as 8-band multispectral images. This imagery comes with the multispectral bands at 1.2 m resolution and the panchromatic band at 50cm resolution. We also trialled imagery from the WorldView3 satellite that has a spatial resolution of 30 cm per pixel in the panchromatic band.

The images were sourced from the DigitalGlobe search and discovery tool: https://discover.digitalglobe.com/ . This tool enables the researcher to filter the type of imagery and dates of interest for the area they request. The images contained in this browser are archival imagery, for new acquisitions contact Digital Globe directly through the MAXAR website: <https://www.digitalglobe.com/contact/customer-service> to order you will need to provide an AOI (area of interest) either as a GIS shapefile or a Google Earth KML file, preferably in a geographic projection. There are limits to the size and shape complexity of the shape that can be ordered which should be discussed with digital globe. Note that there is a delay of several days between ordering and the imagery being acquired, this depends upon the satellite orbit, cloud cover and other tasking.
