# Supplementary material for: Using remote sensing to detect whale strandings in remote areas: The case of sei whales mass mortality in Chilean Patagonia
Source: PLoS One. 2019 Oct 17;14(10):e0222498. doi: 10.1371/journal.pone.0222498 (PMC6797088; doi:10.1371/journal.pone.0222498)
Supplement: S2 Text — (DOCX) [file pone.0222498.s003.docx]

S2 Cost of satellite imagery

Below is a short discussion of the cost of satellite imagery.

One of the advantages of satellite data is that it can have regular repeat acquisition and has a limited bureaucracy (other than the order process). At the time of submitting this paper:

**Archival imagery** of 50cm colour (including 4 band near infrared) imagery is $17.50 per km2. Research/Education users get a 30% reduction on this. NGOs get 5%. US federal Government users get 50% discount. The base price for higher resolution imagery is more so 30cm imagery is $22.50. The minimum area that can be ordered from the archive is 25 km^2^.

**New tasking** is more expensive; $27.50 for 50 cm and $32.50 for 30 cm. The minimum area is also larger at 100 km^2^. Note also that there are some areas of the world (about 10%), classed as high demand countries, where imagery is charged at a higher (+80%) rate – see DigitalGlobe website for more details.

Notes on purchasing:

1. Cloud: the purchaser does not have to acquire imagery with more than 20% cloud and may get a discount if they accept cloudy images. When tasked the satellite provider will continue taking imagery until a non-cloudy image is acquired.
2. Sea-state: At present there is no consideration for sea-state. For offshore images, the purchaser will have to accept a tasked, cloud-free image regardless of the sea-state.

**Satellite or aerial imagery?**

Comparing satellite imagery costs to aerial survey is difficult, as the cost of aerial survey varies depending upon location. From enquires that we have made, aerial survey in some areas is comparable or, in certain cases, cheaper than the price of 30cm imagery, but the cheaper cost of aerial survey may not include all set-up costs and will only be true in a limited number of regions. Satellite imagery is more efficient in remote areas, areas of difficult access (such as disputed territories and open ocean), and does not require special permits or licenses. For areas far from airstrips, satellite imagery may be the only viable alternative to an expensive ship-borne helicopter survey.

Acquisition of satellite imagery is also risk-free and causes zero disturbance, however it does have a lower resolution than aerial imagery. For a large beached whale satellite imagery of 30cm resolution usually will give enough detail, but for smaller whale species (<5m) the resolution of satellites may still not be high enough to discern or count individual strandings.

There may also be the possibility of acquiring free archival imagery of certain areas from DigitalGlobe. This is under discussion at the time of submission.
